# The Parkinson’s phenome—traits associated with Parkinson’s disease in a broadly phenotyped cohort

**DOI:** 10.1038/s41531-019-0077-5

**Published:** 2019-03-27

**Authors:** Karl Heilbron, Alastair J. Noyce, Pierre Fontanillas, Babak Alipanahi, Mike A. Nalls, The 23andMe Research Team, M. Agee, A. Auton, R. K. Bell, K. Bryc, S. L. Elson, N. A. Furlotte, D. A. Hinds, J. C. McCreight, K. E. Huber, A. Kleinman, N. K. Litterman, M. H. McIntyre, J. L. Mountain, E. S. Noblin, C. A. M. Northover, S. J. Pitts, J. F. Sathirapongsasuti, O. V. Sazonova, J. F. Shelton, S. Shringarpure, C. Tian, J. Y. Tung, V. Vacic, C. H. Wilson, Paul Cannon

**Affiliations:** 1 23andMe, Inc., 899W Evelyn Avenue, Mountain View, CA 94041 USA; 2 Preventive Neurology Unit, Wolfson Institute of Preventive Medicine, Queen Mary University of London, London, UK; 3 Department of Clinical and Movement Neurosciences, UCL Institute of Neurology, Queen Square, London, UK; 4 Data Tecnica International, Glen Echo, MD USA; 5 Laboratory of Neurogenetics, National Institute on Aging, Bethesda, USA

## Abstract

In order to systematically describe the Parkinson’s disease phenome, we performed a series of 832 cross-sectional case-control analyses in a large database. Responses to 832 online survey-based phenotypes including diseases, medications, and environmental exposures were analyzed in 23andMe research participants. For each phenotype, survey respondents were used to construct a cohort of Parkinson’s disease cases and age-matched and sex-matched controls, and an association test was performed using logistic regression. Cohorts included a median of 3899 Parkinson’s disease cases and 49,808 controls, all of European ancestry. Highly correlated phenotypes were removed and the novelty of each significant association was systematically assessed (assigned to one of four categories: known, likely, unclear, or novel). Parkinson’s disease diagnosis was associated with 122 phenotypes. We replicated 27 known associations and found 23 associations with a strong a priori link to a known association. We discovered 42 associations that have not previously been reported. Migraine, obsessive-compulsive disorder, and seasonal allergies were associated with Parkinson’s disease and tend to occur decades before the typical age of diagnosis for Parkinson’s disease. The phenotypes that currently comprise the Parkinson’s disease phenome have mostly been explored in relatively small purpose-built studies. Using a single large dataset, we have successfully reproduced many of these established associations and have extended the Parkinson’s disease phenome by discovering novel associations. Our work paves the way for studies of these associated phenotypes that explore shared molecular mechanisms with Parkinson’s disease, infer causal relationships, and improve our ability to identify individuals at high-risk of Parkinson’s disease.

## Introduction

The clinical diagnosis of PD is based on its canonical motor symptoms, but there has recently been a growing understanding of the importance of early non-motor aspects of PD such as anxiety, constipation, and fatigue.^1^ In addition to motor and non-motor prodromal symptoms, epidemiological studies have uncovered a collection of traits, environmental exposures, and comorbidities (we will collectively refer to these as “phenotypes”) that are associated with PD, including reduced coffee consumption, reduced tobacco use, reduced serum uric acid levels, and increased rates of melanoma.^2^ However, the prevalence of PD is approximately 1% in individuals over 65 years old^3^ making it difficult to amass the large numbers of cases required to detect more subtle associations using traditional observational study methods.

Large electronic databases of medical records, billing codes, insurance claims, and prescriptions have enabled studies with larger sample sizes and a wider diversity of phenotypes under consideration.^4,5^ However, these databases are often restricted to billable events and may not capture phenotypes related to sub-clinical symptoms, behavior, lifestyle, morphology, or over-the-counter medication.

Here, we sought to describe the broad spectrum of phenotypes associated with PD—the PD phenome—using a cross-sectional database that included 13,546 PD cases, >1.3 million controls, and 832 phenotypes including diagnoses, family history, medication usage, environmental exposures, and behaviors. We performed association tests using logistic regressions on age-matched and sex-matched case–control cohorts. We used literature reviews to classify significant associations into four categories: known, likely, unclear, and novel associations. Although cross-sectional data are not well-suited for inferring causal relationships, we discuss how our hypothesis-generating approach has meaningfully extended our understanding of the PD phenome.

## Results

### Demographics

Table 1 shows demographic descriptive statistics for cohorts of up to 13,196 PD cases and 148,176 age-matched and sex-matched controls. Cases and controls were similar with respect to age, sex, education, and income index. We found larger differences for body mass index (BMI) and tobacco use. The median BMI of PD cases was 3.1% lower than that of controls and PD cases were less likely to have ever smoked more than 100 cigarettes (cases: 38.4%, controls: 46.8%). Since different subsets of 23andMe research participants responded to each survey question, we constructed separate matched case–control cohorts for each of the 832 phenotypes. Demographic differences between cases and controls across cohorts were similar to the differences in the cohort containing all PD cases shown in Table 1 (Supplementary Data 1).

**Table 1:** Demographics of PD cases and controls

	Cases		Controls		*P*
		N		N	
Age, mean (SD), years	69.8 (11.2)	13,196	69.3 (11.4)	131,960	3.10 × 10^−6^
Female, no. (%)	5,126 (38.8%)	13,196	51,260 (38.8%)	131,960	1
Education level, no. (%)					2.27 × 10^−5^
Less than high school	93 (1.1%)	8,159	1,537 (1.2%)	130,544	
High school diploma	697 (8.5%)	8,159	12,198 (9.3%)	130,544	
Associate’s degree/vocational degree/some college	1,954 (23.9%)	8,159	33,909 (26.0%)	130,544	
Bachelor’s degree	2,391 (29.3%)	8,159	36,276 (27.8%)	130,544	
Master’s/professional degree	2,475 (30.3%)	8,159	37,830 (29.0%)	130,544	
Doctorate degree	549 (6.7%)	8,159	8794 (6.7%)	130,544	
Income index^a^, median (IQR), US dollars	$66,486 ($36,937)	6,872	$66,489 ($37,023)	109,952	3.06 × 10^−1^
Body mass index, median (IQR), kg/m^2^	25.8 (6.1)	10,584	26.6 (6.1)	148,176	8.31 × 10^−81^
Tobacco use, no. (%)	4,085 (38.4%)	10,630	64,719 (46.8%)	138,190	1.21 × 10^−63^

*SD* standard deviation, *IQR* interquartile range, *N* total number of individuals in a cohort

^a^Each individual was assigned an income index value equal to the median household income in the past 12 months in his or her self-reported zip code using data from the U.S. Census Bureau’s most recent (2016) American Community Survey 5-Year Estimates

### Overview of significant associations

**Fig. 1:**
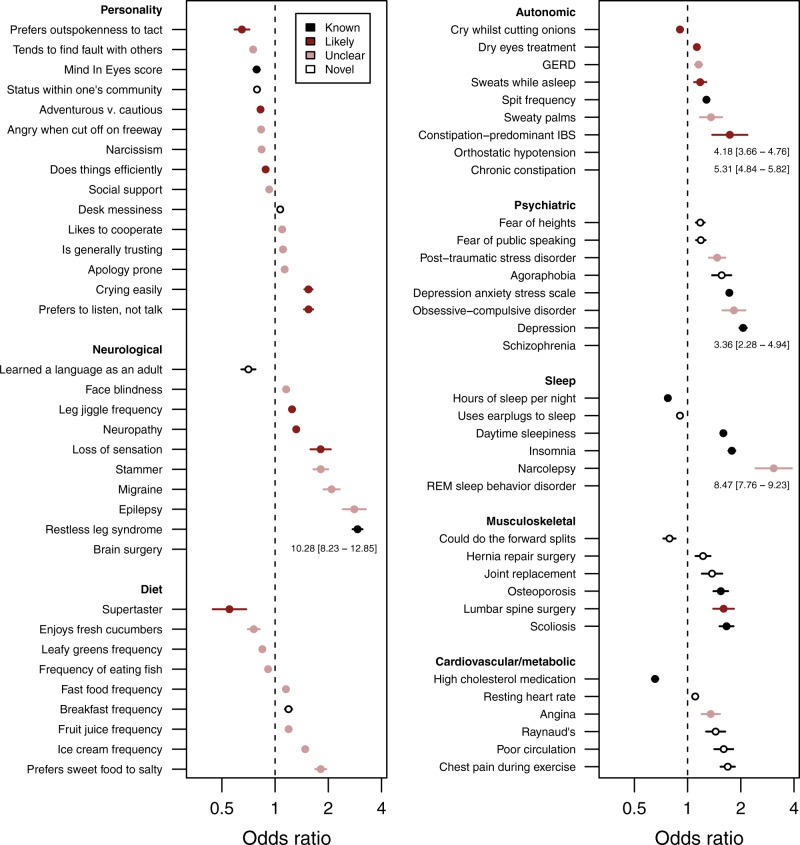
Forest plot of odds ratios between Parkinson’s disease and associated phenotypes. Forest plot of the odds ratios (ORs) ± 95% confidence intervals (CIs) for 69 phenotypes from the following groups that were significantly associated with PD: personality, neurological, diet, autonomic, psychiatric, sleep, musculoskeletal, or cardiovascular/metabolic. Colors denote the extent to which an association between PD and the phenotype had previously been studied. If the upper 95% CI of an association was greater than four, data are presented as text. The dashed line represents an OR of one—no association with PD. To help clarify the directionality of associations, comprehensive phenotype definitions can be found in the Supplementary Data 2. GERD, gastro-esophageal reflux disease; IBS, irritable bowel syndrome; REM, rapid eye movement

**Fig. 2:**
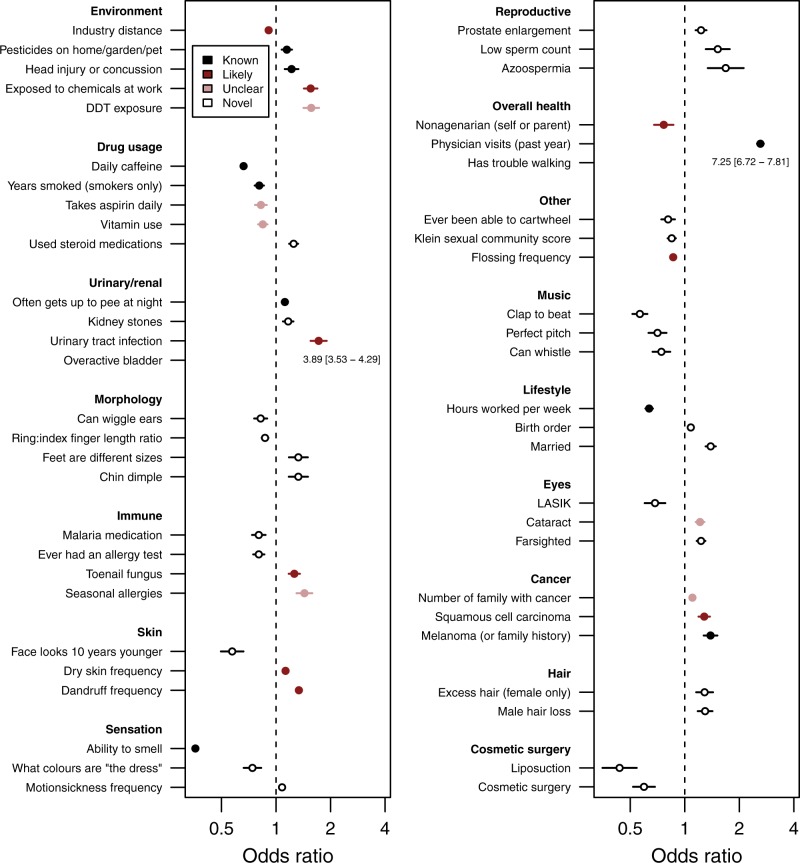
Forest plot of odds ratios between Parkinson’s disease and associated phenotypes. Forest plot of the odds ratios (ORs) ± 95% confidence intervals (CIs) for 53 phenotypes from the following groups that were significantly associated with PD: environment, drug usage, urinary/renal, morphology, immune, skin, sensation, reproductive, overall health, other, music, lifestyle, eyes, cancer, hair, or cosmetic surgery. Colors denote the extent to which an association between PD and the phenotype had previously been studied. If the upper 95% CI of an association was greater than four, data are presented as text. The dashed line represents an OR of one—no association with PD. To help clarify the directionality of associations, comprehensive phenotype definitions can be found in the Supplementary Data 2. DDT, dichlorodiphenyltrichloroethane; LASIK, laser-assisted in situ keratomileusis

We performed age-matched and sex-matched logistic regressions between PD and 832 phenotypes, 436 of which were not highly correlated with each other. Of these 436 associations, 132 were statistically significant after Bonferroni correction (*P* < 6.01 × 10^−5^), 122 of which remained significant after adjusting for education, income index, BMI, and tobacco use (Figs. 1, 2). We were able to replicate 27 “known” associations (see Methods) that have previously been reported in the literature. For example, we replicated the negative association between caffeine intake and PD (OR = 0.662 [95% CI 0.641–0.684], *P* *=* 3.60 × 10^−136^), and the positive association between PD and constipation (OR = 5.31 [95% CI 4.84–5.82], *P* *=* 6.19 × 10^−272^). The size of our cohort and the diversity of phenotypes studied also allowed us to discover 42 novel associations that have not been previously reported. We found 23 phenotypes that were strongly related to known aspects of PD (“likely”) and 30 “unclear” phenotypes where an association with PD has previously been studied, but did not meet our criteria for being classified as “known” or “likely”.

### Comparison with published associations

We investigated whether there were any known associations that our analysis failed to detect. We compared our results with those from a systematic review and meta-analysis of putative PD risk factors^6^ (Supplementary Table 1). The comparison study found 19 significant phenotypes, 10 of which had an analogous phenotype in our PD phenome study. Our study successfully replicated all 10 associations (*P* < 5 × 10^−3^ and concordant direction of effect). For example, we replicated known negative associations with hypertension (OR = 0.863 [95% CI 0.825–0.902], *P* = 9.20 × 10^−11^) and alcohol consumption (for a 1 SD increase: OR = 0.835 [95% CI 0.802–0.870], *P* = 6.33 × 10^−18^). We found that PD was associated with living nearer to a farm (for a 1 SD increase in distance: OR = 0.941 [95% CI 0.911–0.972], *P* *=* 2.22 × 10^−4^), but this narrowly missed our study-wide significance threshold of 6.01 × 10^−5^. The comparison study also highlighted 11 phenotypes that were not significantly associated with PD—possibly due to insufficient power—and 7 of them had an analogous phenotype in our PD phenome study. The direction of effect was similar in both studies for all phenotypes except for aspirin use (23andMe study: OR = 0.825 [95% CI 0.766–0.889], comparison study: OR = 1.11 [95% CI 0.93–1.32]).

## Discussion

Although Parkinson’s disease is diagnosed on the basis of motor signs, the PD phenome is known to encompass a broad range of non-motor features such as autonomic dysfunction, sleep disturbances, cognitive dysfunction, and psychiatric disorders. We systematically tested 832 phenotypes for an association with PD and found 122 significant associations. Some associations represent known consequences of PD symptoms (e.g., trouble walking, more frequent physician visits), known consequences of PD treatment (e.g., having had brain surgery, daytime sleepiness due to dopaminergic medication^7^), or plausible consequences of later-stage PD (e.g., flossing less frequently, less able to clap to a beat). Other associated phenotypes are used in the International Parkinson and Movement Disorder Society research criteria for prodromal Parkinson’s disease^8^ (e.g., REM sleep behavior disorder, olfactory loss). We also identified associations where the underlying causal relationship is unknown. We have organized PD-associated traits into functionally related groups and discuss some of the main findings here.

Several neurological disorders were associated with PD including restless leg syndrome (RLS), migraine, and epilepsy. RLS is more prevalent in people with PD, although previous prevalence estimates have ranged widely and it is unclear whether RLS is a risk factor for PD.^9^

We observed that migraine was positively associated with PD. Only two cohort studies have previously been conducted, but both found that midlife migraine was associated with increased risk of PD.^10,11^ Both studies corrected for head trauma and cardiovascular risk factors and disease since they are risk factors for secondary parkinsonism. We found that most cardiovascular variables were negatively associated with PD (e.g., high cholesterol, BMI, tobacco use, type 2 diabetes) with the exception of angina and chest pain during exercise. We found that the association between PD and migraine remained significant (OR = 1.756 [1.554–1.985], *P* = 1.93 × 10^−19^) after correcting for head injury, angina, and chest pain during exercise using dummy variable imputation. Since the average age of onset is typically much earlier for migraine than for PD, migraine may be a novel PD risk factor.

People with PD were more likely to self-report having been diagnosed with, or treated for, epilepsy or seizures. A recent British study found a significantly greater incidence rate ratio of epilepsy in 23,086 PD cases and 92,343 matched controls, suggesting that PD may be a risk factor for epilepsy.^12^

Psychiatric manifestations are common in PD. Questions pertaining to depression and anxiety have been incorporated into the MDS-UPDRS^1^ and psychosis has been found in up to 60% of PD patients.^13,14^ There has been substantial interest in a connection between obsessive-compulsive disorder (OCD) and PD since both can be treated via deep brain stimulation, can be caused by viral encephalitis, and may involve dysfunction of the basal ganglia.^15^ Kummer and Teixeira^15^ reviewed 11 studies of OCD and OCD symptoms (OCS) in relation to PD and found several methodological issues including small sample sizes (20–124 PD cases) leading to a lack of statistical power. Indeed, four of the five case-control studies assessed yielded non-significant results. With 10,437 PD cases in this particular regression, our study was well-powered to detect a positive association between PD and OCD (*P* = 2.34 × 10^−15^).

People with PD tended to report being less quick to anger, less outspoken, and less talkative. Our results are qualitatively similar to previous observations, which have found that people with PD tended to be less aggressive,^16^ more inhibited,^17^ and more neurotic and introverted.^18^ Whether these personality differences exist prior to PD diagnosis is controversial and may be due to recall bias.^19^

People with PD were more likely to report a preference for, and increased consumption of, sweet foods and beverages. There have been few consistent associations between PD and the intake of specific food items with the exception of coffee^6^ and perhaps dairy products.^20^ However, a recent Italian study found a greater intake of sweets in 600 people with PD compared to 600 age-matched and sex-matched controls.^21^ A German case–control study including 342 people with PD reported a greater intake of sweets, cookies, and cakes.^22^ This was associated with time since diagnosis, suggesting that altered food preferences may be a feature of later stage PD.

Being married was associated with an increased odds of PD. Compared to people who reported never having been married and after adjusting for covariates including age, the odds of PD were: (1) highest in people who were currently married (OR = 1.793 [1.609–1.999], *P* = 5.04 × 10^−26^), (2) attenuated in people who were divorced (OR = 1.268 [1.108–1.450], *P* *=* 5.40 × 10^−4^), and (3) abrogated in people who were widowed (OR = 1.029 [0.852–1.241], *P* = 0.771). We ran a phenome analysis with marital status as the outcome variable and found that marriage was largely negatively associated with a wide variety of diseases, with PD being a notable exception (Supplementary Table 2). This remained true when analyzing females and males separately. These results suggest that the association with PD was unlikely to be due to bias from over-reporting of disease history in married individuals, but we cannot completely rule out the possibility that prodromal features of PD like REM sleep behavior disorder may lead spouses to encourage their partners to seek medical consultation.

We found that seasonal allergies were associated with PD. Although the immune system is known to play an important role in PD,^23^ to our knowledge only two studies of PD and allergies have been published. A retrospective case-control study found that PD was positively associated with allergic rhinitis, but not asthma or hayfever.^24^ A study using the Taiwan National Health Insurance Research Database found a positive, but not significant, association between PD and allergic rhinitis (hazard ratio = 1.29 [0.97–1.72]).^25^ We found that PD was also associated with allergies to plants and antibiotics, but not with allergies to food or animals (Supplementary Data 1). Understanding the biological differences between these different groups of allergies may allow us to hone in on specific immunological pathways that contribute to PD risk. Seasonal allergies typically develop early in life, but further research is needed to determine whether having seasonal allergies is a risk factor for PD. If so, it may be possible to manipulate risk using immune-modulating drugs.

Now that we have identified phenotypes that are correlated with PD it is also possible to test whether these phenotypes are causes or consequences of the disease. Longitudinal prospective cohort studies are often used to perform causal inference, but this approach may be less appropriate in PD since neurodegeneration may begin as long as 20 years before PD diagnosis.^26^ Alternatively, Mendelian randomization is a causal inference method that utilizes data from genome-wide association studies (GWASes) and is not hampered by the lengthy prodromal phase of PD.^27^ We have collected genetic data for all individuals used in this study and will be able to explore causal inference in future work.

Even in the absence of a known causal relationship, phenotypes that are correlated with PD can be used to predict PD as long as the phenotype is enriched in PD cases prior to diagnosis. In many cases, modern machine learning algorithms have been remarkably successful in taking a large set of predictors (e.g., phenotypes)—each weakly associated with the outcome (e.g., PD)—and combining them into a single, strong predictive model.^28^ Indeed, several algorithms have been developed to predict the risk of developing PD using data derived from clinical tests, olfaction tests, genotyping arrays, and online tests and surveys.^8,29,30^ Models based on data from online surveys or electronic health records can be used for low-cost population-wide screening, and may be a valuable tool for prioritizing individuals with a high probability of developing PD for research purposes. Identifying high-risk individuals may also be critically important for the development of effective disease modifying therapies for PD. Because a large proportion of the dopaminergic neurons in the substantia nigra have already died by the time PD is typically diagnosed,^31^ it may already be too late to intervene in the pathological cascade. Interventional trials in high-risk individuals may therefore be critical to the development of therapies that slow or halt PD progression.

There were several limitations to our study. First, PD diagnoses were self-reported and not confirmed by a clinician. However, we have previously shown that self-reported PD diagnosis was equivalent to a telemedicine-based diagnosis by a neurologist in 100% of a sample of fifty people with PD from the 23andMe cohort.^32^ Furthermore, our PD cohort has been used extensively in GWAS studies, contributing to the discovery of over 40 PD loci.^33^ However, as misdiagnosis is not uncommon even in a clinical setting,^34^ our study likely includes a unknown proportion of misdiagnosed individuals. Second, our analyses were restricted to individuals of European ancestry and further work is needed to determine whether our results are applicable to individuals from other ethnic backgrounds. Third, as with any epidemiological study, it is always possible that a subset of our associations were driven by unmeasured confounding variables, such as the use of PD medications. However, only 9 associations (6.4%) showed evidence of being confounded by four important demographic variables (education, income index, BMI, and tobacco use), two of which showed relatively large differences between PD cases and controls (BMI and tobacco use). Fourth, case–control studies may be subject to recall bias, although this should be weaker for phenotypes that have not been widely reported to be associated with PD, such as those in the “novel” and “unclear” categories. Fifth, our study lacked temporal data for most phenotypes and we were therefore unable to determine whether they occurred prior to PD diagnosis. Finally, each regression was performed in a separate cohort, limiting our ability to make comparisons between association tests. Despite these limitations, we were able to replicate 27 known associations and—apart from known associations for which we lacked relevant survey data, such as well water use—there was only one known association that we narrowly failed to replicate.

Epidemiological studies have identified a wide range of motor and non-motor phenotypes linked to PD. We successfully reproduced much of the established PD phenome using a systematic screen of a large database of online survey responses. We validated 27 known associations and highlighted an additional 23 associations with a strong prior link to a known association. Of the phenotypes that we tested that are part of the established PD phenome, only one (living near a farm) narrowly failed to reach significance. In addition, our work extends the PD phenome by discovering 42 novel associations and adding support to 30 associations that have previously been studied, but not in sufficient depth to be considered known or likely. Among these 30 unclear associations we found that migraine, OCD, and seasonal allergies were associated with PD, all of which tend to have an age of onset several decades before the average age of diagnosis for PD and may therefore represent PD risk factors. The novel phenotypic associations identified by our hypothesis-generating approach should be followed up in additional case–control studies, as well as in prospective cohort and Mendelian randomization studies. Having a better understanding of the PD phenome may enable further studies that dissect the molecular mechanisms that drive these associations, perform causal inference to identify novel PD risk factors, and allow for the development of low-cost survey-based population screening tools to identify at-risk individuals.

## Methods

### Study participants

People with PD were recruited through a targeted email campaign in conjunction with the Michael J. Fox Foundation, The Parkinson’s Institute and Clinical Center, and many other PD patient groups and clinics. Emails or hard copy mailings were sent to all individuals who had registered with these groups as PD patients. For a fuller description of recruitment, screening, and genotyping see Do et al.^35^ In addition, individuals with PD were identified via online surveys from customers of the personal genetics company, 23andMe, Inc., who had consented to participate in research.

PD cases were individuals who self-reported having been diagnosed with PD. We excluded people if they reported a change in diagnosis or uncertainty about their diagnosis. We have previously shown that self-reported PD case status is approximately as reliable as clinically-diagnosed PD with 50 out of 50 cases confirmed via telemedicine interview.^32^ Age- and sex-matched controls were drawn from 23andMe research participants who self-reported that they have not been diagnosed with PD. We removed individuals who self-reported having ever been diagnosed with: (1) an atypical parkinsonism (e.g., dementia with Lewy bodies, progressive supranuclear palsy, multiple system atrophy, corticobasal degeneration) or a non-parkinsonian tremor disorder; or (2) stroke, deep vein thrombosis, or pulmonary embolism (to reduce the probability of including individuals with vascular parkinsonism).

### Determining unrelated individuals of European ancestry

We focused on European ancestry individuals because 88.9% of all people with PD in the 23andMe database had >97% European ancestry and because PD incidence and prevalence may differ by ethnicity.^36,37^ All study participants were genotyped by 23andMe and ancestry composition was performed as previously reported.^38^ A maximal set of unrelated individuals was chosen for the analysis using a segmental identity-by-descent estimation algorithm.^39^ Individuals were defined as related if they shared 700 cM identity-by-descent, including regions where the two individuals share either one or both genomic segments identical-by-descent. This level of relatedness (roughly 20% of the genome) corresponds approximately to the minimal expected sharing between first cousins in an outbred population.

### Protocol approvals, registrations, and patient consents

All individuals provided written informed consent, and 23andMe’s human subjects protocol was reviewed and approved by Ethical & Independent Review Services, an AAHRPP-accredited institutional review board. This study was conducted according to the principles set out in the Declaration of Helsinki.

### Phenotypic data

Phenotypic data (including PD status) were collected using web or mobile-based surveys. Surveys were either developed by 23andMe scientists or based on existing instruments published in the medical literature. All phenotypes for which we have conducted a genome-wide association study since January 1st, 2016 were included. In addition, we included environmental exposures that are unlikely to have a strong genetic component, such as pesticide use. We excluded phenotypes related to covariates used in downstream analyses, drug efficacy or side effects, and phenotypes for which PD was an inclusion or exclusion criterion. We excluded phenotypes for which there were fewer than 2000 PD cases or fewer than 10,000 total individuals who had answered the survey questions necessary to define the phenotype. We removed case-control phenotypes if there were fewer than 30 cases who were also PD cases. Finally, we excluded phenotypes where we could not assign at least one age-matched and sex-matched control to each PD case. Phenotype definitions are provided for phenotypes that were significantly associated with PD (Supplementary Data 2). Analyses were run on phenotypic data collected between November 10th, 2007 and October 10th, 2017.

### Age-matched and sex-matched controls

For each of the 832 phenotypes, we constructed a case–control cohort including all PD cases who had responded to the survey question(s) used to define the phenotype. We then selected as many age-matched and sex-matched controls for each case as possible. Specifically, we (1) separated cases by biological sex and divided each into ten evenly-populated age bins; (2) determined the number of controls that fell into each corresponding sex/age bin; (3) divided the number of controls in a bin by the number of corresponding cases; (4) determined the minimum number of available controls per case across all bins, a number which we refer to here as *n*; and (5) randomly selected *n* age-matched and sex-matched controls for each case.

### Identifying significant associations

For each of the 832 phenotypes, we performed a logistic regression using R (version 3.2.2) with PD as the dependent variable and the phenotype in question as the independent variable. All regressions were run using a standard set of covariates: age, sex, date of entry into the research cohort (split into 20 evenly populated bins, used to correct for differences in demographics and available surveys over time), and the first five genetic principal components (used to correct for differences in ancestry). In order to focus on phenotypes that were not highly correlated with each other, we computed the partial correlation coefficient between each possible pair of phenotypes, a value ranging between −1 (perfectly anticorrelated) to 1 (perfectly correlated). For each pair of phenotypes where the absolute value of the partial correlation coefficient was greater than 0.25, we removed the phenotype that was less strongly associated with PD based on *P* value. To mitigate the risk of spurious associations with PD arising through multiple comparisons, we imposed a Bonferroni-based *P* value threshold based on all 832 regressions performed in this study (6.01 × 10^−5^).

### Correcting for demographic variables

To test whether significant associations may have been driven by demographic differences between PD cases and controls, we re-ran regressions including education, income index, body mass index (BMI) and tobacco use as covariates. Income index was defined as the median household income in the past 12 months in an individual’s self-reported zip code using data from the U.S. Census Bureau’s most recent (2016) American Community Survey 5-Year Estimates. For the minority of individuals who had not answered the survey questions used to define a given demographic variable, we imputed data using dummy variable imputation. Across all regressions, the median percentages of imputed data were 18% for education, 6% for tobacco, 4% for BMI, and 22% for income index. Phenotypes that no longer passed our significance threshold (*P* > 6.01 × 10^−5^) were removed from subsequent analyses.

### Classification of associations based on previous literature

We used PubMed to perform a MEDLINE database search using the keyword “Parkinson” and keywords to describe each significant phenotype. We supplemented these PubMed searches with simple web searches. We identified meta-analyses, review articles, cohort studies, and case–control studies that tested for an association between the prevalence (or incidence) of PD and the prevalence (or incidence) of the phenotype in question. We extracted information regarding these associations from publications that met our search criteria. For each association, one of the authors (KH) manually assessed the results from all relevant publications and assigned the association to one of four categories denoting the extent to which the association has previously been studied: known, likely, unclear, and novel. Another author (AJN) reviewed these assignments and discrepancies in assignments between the authors were discussed until consensus was reached.

An association was classified as “known” if: (1) the phenotype is used in the Movement Disorder Society (MDS) criteria for PD^40^ or prodromal PD,^8^ or the MDS-sponsored revision of the Unified Parkinson’s Disease Rating Scale (MDS-UPDRS)^1^; (2) a meta-analysis has found a significant association; or (3) a review article by domain experts asserted that an association is known. “Likely” associations were those where we were unable to find previous literature that demonstrated an association, but where we achieved consensus agreement that the phenotype was strongly related to “known” aspects of PD. “Unclear” associations were those that have been previously been reported in the literature, but did not meet the requirements for the “known” or “likely” categories. “Novel” associations were those for which we could not find any relevant literature.

### Diseases associated with marriage

We repeated our phenome analysis using marital status as the dependent variable. Rather than use age-matched and sex-matched controls, however, we included five additional age covariates: age raised to the power of two to age raised to the power of six.

### Previous publication

This article has previously been published as a preprint: 10.1101/270934

### Reporting Summary

Further information on experimental design is available in the Nature Research Reporting Summary linked to this article.

## Supplementary information

Supplementary Information.

Reporting Summary

Supplementary Data 1.

Supplementary Data 2.

## Data Availability

Model outputs for all 832 logistic regressions are provided as Supplementary Information.

## Supplementary information

**Supplementary information** accompanies the paper on the *npj Parkinson’s Disease* website (10.1038/s41531-019-0077-5).
