# Supplementary material for: The Parkinson’s phenome—traits associated with Parkinson’s disease in a broadly phenotyped cohort
Source: NPJ Parkinsons Dis. 2019 Mar 27;5:4. doi: 10.1038/s41531-019-0077-5 (PMC6437217; doi:10.1038/s41531-019-0077-5)
Supplement: Supplementary file 1 — Supplementary Information. [file 41531_2019_77_MOESM1_ESM.pdf]

Supplementary Table 1: Comparison with published associations

Noyce et al. 2012 meta-analysis		23andMe	
Phenotype	OR/RR (95% CI)	Phenotype	OR (95% CI)
Significant negative associations			
Smoking (current v. never)	0.44 (0.39 - 0.50)	Smoking (current v. never)	0.64 (0.56 - 0.72)
Smoking (ever v. never)	0.64 (0.60 - 0.69)	Smoking (ever v. never)	0.81 (0.77 - 0.84)
Coffee	0.67 (0.58 - 0.76)	Daily caffeine	0.66 (0.64 - 0.68)
Hypertension	0.74 (0.61 - 0.90)	Hypertension	0.86 (0.83 - 0.90)
Alcohol consumption	0.90 (0.84 - 0.96)	Alcoholic drinks per week	0.84 (0.80 - 0.87)
Smoking (previous v. never)	0.78 (0.71 - 0.85)	No analogous phenotype	
NSAIDs	0.83 (0.72 - 0.95)	No analogous phenotype	
Calcium channel blockers	0.90 (0.82 - 0.99)	No analogous phenotype	
Significant positive associations			
Constipation	2.34 (1.55 - 3.53)	Chronic constipation	5.31 (4.84 - 5.82)
Mood disorder	1.86 (1.64 - 2.11)	Depression	2.06 (1.97 - 2.16)
Pesticides	1.78 (1.50 - 2.10)	Pesticides on home/garden/pet	1.15 (1.08 - 1.22)
Head injury	1.58 (1.30 - 1.91)	Head injury or concussion	1.22 (1.12 - 1.33)
Rural living	1.43 (1.13 - 1.81)	Living distance from a farm	1.06 (1.03 - 1.10)
Family history of PD (any)	4.45 (3.39 - 5.83)	No analogous phenotype	
Family history of PD (1st degree)	3.23 (2.65 - 3.93)	No analogous phenotype	
Family history of tremor	2.74 (2.10 - 3.57)	No analogous phenotype	
Beta blockers	1.28 (1.19 - 1.39)	No analogous phenotype	
Farming/agriculture	1.26 (1.10 - 1.44)	No analogous phenotype	
Well water	1.21 (1.04 - 1.40)	No analogous phenotype	
Non-significant associations			
Oral contraceptives	0.73 (0.43 - 1.25)	Currently using birth control	0.83 (0.66 - 1.05)
Statins	0.79 (0.61 - 1.02)	High cholesterol medication	0.65 (0.63 - 0.68)
Hormone replacement therapy	0.90 (0.67 - 1.21)	Hormone replacement therapy	0.97 (0.89 - 1.06)
Diabetes	0.91 (0.72 - 1.15)	Type 2 diabetes	0.79 (0.74 - 0.85)
Tea	1.00 (0.72 - 1.38)	Daily caffeine from tea	0.95 (0.92 - 0.98)
Aspirin	1.11 (0.93 - 1.32)	Takes aspirin daily	0.83 (0.77 - 0.89)
Ulcers	1.37 (0.36 - 5.31)	Ulcers	1.18 (1.08 - 1.30)
Oophorectomy	0.76 (0.52 - 1.13)	No analogous phenotype	
Cancer	1.01 (0.94 - 1.09)	No analogous phenotype	
Acetominophen/paracetamol	1.02 (0.76 - 1.36)	No analogous phenotype	

General anesthetic	1.10 (0.77 - 1.58)	No analogous phenotype

Noyce *et al.* (2012) performed a systematic review and meta-analysis of studies that tested for an association between Parkinson's disease (PD) and putative PD risk factors. They found 19 putative risk factors that were significantly associated with PD. The 19 odds ratios (ORs) or relative risks (RRs) from the original publication are presented and compared to the ORs that we have calculated using analogous phenotypes from our analysis of the PD phenome. All phenotypes from the Noyce *et al.* study are binary variables (presence/absence). Three phenotypes from our 23andMe PD phenome study are continuous variables (daily caffeine, alcoholic drinks per week, living distance from a farm), and ORs reflect a one standard deviation increase in the phenotype. CI = confidence interval, NSAID = non-steroidal anti-inflammatory drug, CCB = calcium channel blocker.

Supplementary Table 2: Diseases associated with marriage

Disease	Odds ratio (95% CI)	P	N
Negative associations			
Anxiety	0.73 (0.72 - 0.74)	$<1x10^{-300}$	1,018,826
Post-traumatic stress disorder	0.59 (0.58 - 0.61)	$<1x10^{-300}$	1,020,115
Insomnia	0.74 (0.74 - 0.75)	$<1x10^{-300}$	1,033,720
ADHD	0.69 (0.68 - 0.70)	$<1x10^{-300}$	1,020,090
Bipolar disorder	0.58 (0.57 - 0.59)	$<1x10^{-300}$	1,025,472
Chronic pain	0.78 (0.77 - 0.79)	$3.71x10^{-181}$	947,650
COPD	0.59 (0.57 - 0.62)	$8.47x10^{-146}$	972,336
Autism spectrum disorder	0.45 (0.42 - 0.48)	$4.27x10^{-127}$	1,027,792
Schizophrenia	0.27 (0.24 - 0.30)	$3.30x10^{-123}$	1,027,486
Obsessive-compulsive disorder	0.73 (0.71 - 0.75)	$5.91x10^{-115}$	1,023,031
Chronic bronchitis	0.65 (0.62 - 0.67)	$4.84x10^{-108}$	970,261
Eating disorder	0.75 (0.73 - 0.77)	$5.46x10^{-96}$	1,015,288
Agoraphobia	0.81 (0.80 - 0.83)	$4.36x10^{-74}$	600,849
Phobia	0.72 (0.69 - 0.74)	$2.12x10^{-71}$	1,016,141
Osteoporosis	0.86 (0.84 - 0.87)	$6.11x10^{-68}$	986,861
Alcoholism	0.54 (0.50 - 0.58)	$2.02x10^{-60}$	57,966
Type 2 diabetes	0.86 (0.85 - 0.88)	$4.14x10^{-58}$	757,838
Seasonal affective disorder	0.85 (0.83 - 0.86)	$3.37x10^{-57}$	188,843
Adult-onset asthma	0.87 (0.85 - 0.88)	$1.37x10^{-56}$	904,222
Carpal tunnel syndrome	0.88 (0.87 - 0.90)	$9.91x10^{-54}$	984,732
Immunodeficiency	0.82 (0.80 - 0.84)	$2.82x10^{-52}$	381,493
Osteoarthritis	0.90 (0.89 - 0.91)	$4.19x10^{-50}$	984,011
Memory loss	0.75 (0.73 - 0.78)	$1.38x10^{-44}$	439,506
Scoliosis	0.87 (0.85 - 0.89)	$1.22x10^{-43}$	973,336
Positive associations			
In vitro fertilization	2.34 (2.19 - 2.50)	$1.45x10^{-139}$	257,420
High cholesterol medication use	1.14 (1.13 - 1.16)	$4.97x10^{-103}$	1,064,147
Basal cell carcinoma	1.20 (1.18 - 1.22)	$1.67x10^{-85}$	1,017,944
Unexplained infertility	2.06 (1.88 - 2.25)	$3.14x10^{-56}$	64,918
Actinic keratosis	1.19 (1.16 - 1.22)	$4.50x10^{-45}$	1,015,071
Parkinson's disease	1.51 (1.42 - 1.60)	$5.85x10^{-42}$	990,427

Marital status phenome results for diseases (excluding infections and injuries) with a P value less than that for Parkinson's disease and marriage ($5.85x10^{-42}$, shown at the bottom of the table). CI = confidence interval, N = total number of individuals used in each regression, ADHD = attention deficit hyperactivity disorder, COPD = chronic obstructive pulmonary disease.
