# Supplementary material for: The Parkinson’s phenome—traits associated with Parkinson’s disease in a broadly phenotyped cohort
Source: NPJ Parkinsons Dis. 2019 Mar 27;5:4. doi: 10.1038/s41531-019-0077-5 (PMC6437217; doi:10.1038/s41531-019-0077-5)
Supplement: Supplementary file 4 — Supplementary Data 2. [file 41531_2019_77_MOESM4_ESM.docx]

**Phenotype definitions**

**Overview**

Below are phenotype definitions for the 4 demographic phenotypes and 124 significant phenotypes, sorted by *P* value from lowest to highest. For each we provide an internal name, publication name, and definition. The internal name is the identifier used to tag each phenotype in the 23andMe Research Environment. The publication name is the identifier used to represent each phenotype throughout this study and is intended to be short and intuitive. The definition section describes how each phenotype was defined. Where relevant, we have included all or a subset of possible responses in parentheses at the end of the definition. If there were three or fewer possible responses, all are listed separated by commas. If there were greater than three possible responses (typically five), the most extreme options are presented separated by a hyphen.

**Sociodemographic phenotypes**

**Internal name:** bmi

**Publication name:** Body mass index

**Definition:** Mass (kilograms) divided by height squared (meters^2^)

**Internal name:** ever_tobacco_user

**Publication name:** Tobacco use

**Definition:** Cases have smoked at least 100 cigarettes, controls have not.

**Internal name:** education_years

**Publication name:** Education

**Definition:** “What is the highest degree or level of school you have completed? If currently enrolled, please select the previous grade or highest degree received” (less than high school, high school diploma or equivalency [GED], Associate’s degree [for example, AA, AS], Vocational degree, some college but no degree, Bachelor’s degree [for example, BA, BS], Master’s degree [for example, MA, MS, MEng, MEd, MSW, MBA], Professional degree beyond a Bachelor’s degree [for example, MD, DDS, DVM, LLB, JD], doctoral degree [for example, PhD, EdD])

**Internal name:** income_index

**Publication name:** Income index

**Definition:** Each individual was assigned an income index value equal to the median household income in the past 12 months in his or her self-reported zip code using data from the U.S. Census Bureau’s most recent (2016) American Community Survey 5-Year Estimates

**Significant phenotypes**

**Internal name:** iqb.ability_to_smell

**Publication name:** Ability to smell

**Definition:** “How would you rate your current ability to smell?” (very poor – very good)

**Internal name:** iqb.trouble_walking

**Publication name:** Has trouble walking

**Definition:** “Do you have trouble walking for any reason” (yes, no, I’m not sure)

**Internal name:** iqb.rem_sleep_behavior_disorder_dx

**Publication name:** REM sleep behaviour disorder

**Definition:** “Have you been diagnosed with rapid eye movement (REM) sleep behavior disorder or been told that you act out your dreams while lying asleep in bed (as opposed to sleepwalking)?” (no, yes, I'm not sure)

**Internal name:** iqb.physician_visits_12mo

**Publication name:** Physician visits (past 12 months)

**Definition:** “How many times have you been to see a physician in the past 12 months, for any health issue?” (0 - 3+)

**Internal name:** iqb.chronic_constipation

**Publication name:** Chronic constipation

**Definition:** “Do you currently suffer from chronic constipation (fewer than 3 bowel movements per week)?” (yes, no, not sure)

**Internal name:** restless_leg_syndrome

**Publication name:** Restless leg syndrome

**Definition:** The 23andMe restless leg syndrome phenotype is an aggregation of three sub-phenotypes, which I will refer to as #1, #2, and #3. Data is taken first from #1. If a person has not been assigned as a case or control via #1, data is taken from #2. Data is taken from #3 last. As of 2017.11.09, the percentage of cases respectively contributed by each sub-phenotype is: 44.8%, 52.6%, and 2.6%. Here are the phenotype definitions for each sub-phenotype:

- HIP_restless_leg_syndrome
  - “Have you ever been diagnosed with, or treated for, restless legs syndrome?” (yes, no, I’m not sure)
- iqb.restless_leg_syndrome
  - “Have you ever been diagnosed with restless leg syndrome?” (yes, no, I’m not sure)
- HIP_sleep_disorder_type_RLS: as above, this sub-phenotype is an aggregation of four sub-sub-phenotypes, which I will refer to as #3a, #3b, #3c, and #3d. As of 2017.11.09, the percentage of cases respectively contributed by each sub-sub-phenotype is: 0.043%, 0.053%, 0.32%, and 2.16%. Their definitions are:
  - health_intake.A_sleep_disorder_type_RLS
    - Research participants were asked “Have you ever been diagnosed with or treated for any of the following conditions?” and then shown a list of various groups of disorders. Those that answered “yes” to “A sleep disorder” were asked “What sleep disorders have you been diagnosed with? Please select all that apply.” and then shown a list of options. Cases selected “Restless leg syndrome”. Controls include both people who do not have a sleep disorder and people who have a sleep disorder other than RLS.
  - health_intake.B_sleep_disorder_type_RLS
    - Research participants were asked “Have you ever been diagnosed with or treated for any of the following conditions? Post-traumatic stress disorder (PTSD), Autism, Asperger's, Sleep disorder”. Those who answered “yes” were asked “Have you ever been diagnosed with or treated for a sleep disorder?”. Those who answered “yes” were asked “What sleep disorders have you been diagnosed with? Please select all that apply.” and then shown a list of various groups of disorders. Those that answered “yes” to “A sleep disorder” were asked “What sleep disorders have you been diagnosed with? Please select all that apply.” and then shown a list of options. Cases selected “Restless leg syndrome”. Controls are people who did not indicate that they have RLS.
  - health_intake.P_sleep_disorder_type_RLS
    - Same as #3b above
  - health_profile.hp_sleep_disorder_type_RLS
    - Same as #3b above

**Internal name:** cognitive_decline

**Publication name:** Cognitive decline

**Definition:** Any report of cognitive impairment or memory loss in individuals aged 65 and older, excluding Alzheimer's disease cases

**Internal name:** dass_any

**Publication name:** Depression anxiety stress scale

**Definition:** Depression Anxiety Stress Scale scores, averaged over 6 questions:

- Worry: “Over the last two weeks, I was worried about situations in which I might panic and make a fool of myself” (Did not apply to me at all – Applied to me very much or most of the time)
- Panic: “Over the last two weeks, I felt I was close to panic” (Did not apply to me at all – Applied to me very much or most of the time)
- Scared: “Over the last two weeks, I felt scared without any good reason” (Did not apply to me at all – Applied to me very much or most of the time)
- Heart: “Over the last two weeks, I was aware of the action of my heart in the absence of physical exertion (for example, sensing a heart rate increase, your heart missing a beat)” (Did not apply to me at all – Applied to me very much or most of the time)
- Breathing_difficulty: “Over the last two weeks, I experienced breathing difficulty (for example, excessively rapid breathing, breathlessness in the absence of physical exertion)” (Did not apply to me at all – Applied to me very much or most of the time)
- Trembling: “Over the last two weeks, I experienced trembling (for example, of your hands)” (Did not apply to me at all – Applied to me very much or most of the time)

**Internal name:** depression

**Publication name:** Depression

**Definition:** Combines multiple questions about depression, including

- “Have you ever been diagnosed with, or treated for, depression?” (yes, no, I’m not sure)
- “Have you ever been diagnosed with clinical depression?” (yes, no, I’m not sure)

**Internal name:** HIP_urinary_condition_type_OAB

**Publication name:** Overactive bladder

**Definition:** “What type of urinary condition were you diagnosed with most recently?” Cases selected “Overactive bladder (OAB) or urge incontinence”, controls did not. From the Mayo Clinic: “Overactive bladder causes a sudden urge to urinate. The urge may be difficult to stop, and overactive bladder may lead to the involuntary loss of urine (urge incontinence).”

**Internal name:** insomnia_broad

**Publication name:** Insomnia

**Definition:** An aggregation of several questions, including “Have you ever been diagnosed with, or treated for, insomnia?” (yes, no, I’m not sure)

**Internal name:** iqb.daytime_sleepiness

**Publication name:** Daytime sleepiness

**Definition:** “How often do you feel sleepy during the daytime?” (never – very often)

**Internal name:** iqb.ice_cream_frequency

**Publication name:** Ice cream frequency

**Definition:** “In a typical week, how often do you eat ice cream (including frozen yogurt and low fat ice cream)?” (none – several times per day)

**Internal name:** iqb.dandruff_frequency

**Publication name:** Dandruff frequency

**Definition:** “How often do you have dandruff? (If you use treatments for your dandruff, please answer for the frequency of occurrence without treatment.)” (never – always or almost always)

**Internal name:** current_caffeine_tx

**Publication name:** Daily caffeine (mg)

**Definition:** Daily mgs of caffeine from coffee/tea/soda/energy drink, transformed by log10(x+75)

**Internal name:** orthostatic_hypotension

**Publication name:** Orthostatic hypotension

**Definition:** Cases: answered “yes” to at least one of several questions pertaining to having orthostatic hypotension

**Internal name:** brain_surgery

**Publication name:** Brain surgery

**Definition:** “Have you ever had any of the following head surgeries?: Brain surgery” (yes, no, I’m not sure)

**Internal name:** work_hours

**Publication name:** Hours worked per week

**Definition:** Hours per week worked

**Internal name:** iqb.cry_easily

**Publication name:** Crying easily

**Definition:** “Do you cry easily?” (yes, no, not sure)

**Internal name:** iqb.neuropathy

**Publication name:** Neuropathy

**Definition:** “How often do you experience tingling in your hands or feet?” (never – very often)

**Internal name:** sweet_v_salty

**Publication name:** Prefers sweet food to salty

**Definition:** People were asked if they preferred sweet food, salty food, both, or neither. Cases said sweet only, controls said salty only.

**Internal name:** leg_jiggle

**Publication name:** Leg jiggle frequency

**Definition:** Leg jiggle, scored 0 (never) to 4 (frequently)

**Internal name:** tends_to_find_fault_with_others

**Publication name:** Tends to find fault with others

**Definition:** "I am someone who tends to find fault with others" (strongly disagree – strongly agree)

**Internal name:** iqb.avg_sleep

**Publication name:** Average hours of sleep per night

**Definition:** “How many hours of sleep do you get on a typical night?” (<4h - >11h)

**Internal name:** epilepsy

**Publication name:** Epilepsy

**Definition:** A combination of many questions about epilepsy, the most common of which is “Have you ever been diagnosed with, or treated for, epilepsy or seizures?” (yes, no, I’m not sure)

**Internal name:** iqb.talk_versus_listen

**Publication name:** Prefers to listen rather than talk

**Definition:** “If forced to choose, would you say you generally prefer to talk or to listen?” (talk, listen, I'm not sure)

**Internal name:** iqb.adventurous

**Publication name:** Adventurous v. cautious

**Definition:** “If forced to choose, would you consider yourself to be more cautious or more adventurous?” (very cautious – very adventurous)

**Internal name:** alzheimers

**Publication name:** Alzheimer’s

**Definition:** A combination of several questions about a physician diagnosis of AD. Cases and controls must be at least 55 years old.

**Internal name:** iqb.spit_frequency

**Publication name:** Spit frequency

**Definition:** “How often do you have the urge to clear your throat and spit?” (less than monthly - multiple times per day)

**Internal name:** iqb.stammer

**Publication name:** Stammer

**Definition:** “Have you ever had a stammer or stutter?” (yes, no, not sure)

**Internal name:** iqb.chest_pain_exercise

**Publication name:** Chest pain during exercise

**Definition:** “Have you ever experienced chest pain during exercise?” (yes, no, not sure)

**Internal name:** iqb.clap_to_beat

**Publication name:** Clap to beat

**Definition:** “Can you clap in time with a musical beat?” (yes, no, not sure)

**Internal name:** high_cholesterol_meds

**Publication name:** High cholesterol medication

**Definition:** Cases: answered “yes” to at least one of several questions pertaining to taking cholesterol-lowering medication

**Internal name:** migraine_ICHD

**Publication name:** Migraine

**Definition:** Migraine diagnosis based on ICHD-II criteria. The criteria for cases are: 1) 5 or more headaches, 2) these attacks must last at least 3-4 hours, 3) 2 out of 4 common migraine symptoms (severity, unilateral, pulsing, and worsened by movement), 4) sensitivity to (light and sound) or nausea, 5) not attributed to another disorder. The criteria for controls are: 1) do not report a migraine diagnosis, and 2) does not satisfy criteria 1-4 for cases. Note: we do not enforce criterion #5 in any real way.

**Internal name:** community_standing

**Publication name:** Status within one’s community

**Definition:** Community standing, assessed with a colored ladder. red=0 is the lowest rung, black=9 is the highest.

**Internal name:** iqb.cut_off_freeway_anger

**Publication name:** Angry when cut off on freeway

**Definition:** “Someone cuts you off on the freeway. How irritated are you?” (not at all - furious)

**Internal name:** scoliosis

**Publication name:** Scoliosis

**Definition:** Cases: answered “yes” to at least one of several questions pertaining to having scoliosis

**Internal name:** iqb.narcissism

**Publication name:** Narcissism

**Definition:** “How narcissistic (a narcissist is someone who is egotistical, self-focused, and vain) do you think that you are?” (not at all - completely)

**Internal name:** mind_in_eyes_qt

**Publication name:** Mind In Eyes score

**Definition:** Number correct on mind in eyes survey, scores from 0–36. This test is a measure of adult "mentalising", the ability to sense other people's emotions based on their facial expressions, and is believed to be an important component of empathy. The test has been used to distinguish between adults with Asperger syndrome, adults with high-functioning autism, and people without these conditions. Scores on this test also correlate with the number of autistic traits in adults of normal intelligence.

**Internal name:** iqb.chemical_exposure_work

**Publication name:** Exposed to chemicals at work

**Definition:** “Have you ever been exposed to hazardous chemicals through your work?” (yes, no, not sure)

**Internal name:** urinary_tract_inf

**Publication name:** Urinary tract infection

**Definition:** Combines multiple questions about ever having been diagnosed with a UTI

**Internal name:** married

**Publication name:** Married

**Definition:** Case: married, excluding widowed. Control: single/partnered/divorced/separated.

**Internal name:** loss_of_sensation

**Publication name:** Loss of sensation

**Definition:** “Have you ever had one of the neurological conditions: loss of sensation?” (yes, no, I'm not sure)

**Internal name:** narcolepsy

**Publication name:** Narcolepsy

**Definition:** Cases: answered “yes” to at least one of several questions pertaining to having narcolepsy

**Internal name:** iqb.ddt_exposure

**Publication name:** DDT exposure

**Definition:** “Have you ever been exposed to DDT?” (yes, no, not sure)

**Internal name:** iqb.fruit_juice_frequency

**Publication name:** Fruit juice frequency

**Definition:** “In a typical week, how often do you drink fruit juice, such as apple or orange juice?” (none - 14+)

**Internal name:** iqb.leafy_greens_frequency

**Publication name:** Leafy greens frequency

**Definition:** “In a typical week, how often do you eat leafy green vegetables, such as lettuce, spinach, or kale?” (0 - 14+)

**Internal name:** iqb.fast_food_frequency

**Publication name:** Fast food frequency

**Definition:** “In a typical week, how often do you eat fast food?” (not at all – several times a day)

**Internal name:** iqb.flossing_frequency

**Publication name:** Flossing frequency

**Definition:** “How often do you floss your teeth?” (less than once a week – several times a day)

**Internal name:** osteoporosis

**Publication name:** Osteoporosis

**Definition:** Cases: answered “yes” to at least one of several questions pertaining to having osteoporosis

**Internal name:** iqb.prosopagnosia

**Publication name:** Face blindness

**Definition:** “How frequently do you have difficulty remembering or recognizing people's faces?” (never - very often)

**Internal name:** ocd

**Publication name:** Obsessive-compulsive disorder

**Definition:** Combines multiple questions about OCD, including “Have you ever been diagnosed with, or treated for, obsessive-compulsive disorder (OCD)?” (yes, no, I’m not sure)

**Internal name:** iqb.tact_versus_outspokenness

**Publication name:** Prefers outspokenness to tact

**Definition:** “Which of the following do you value more highly?” (To be tactful and considerate of other people's feelings, To speak your mind, I’d rather not say). Cases = outspoken, controls = tact.

**Internal name:** iqb.dry_skin_frequency

**Publication name:** Dry skin frequency

**Definition:** “How often do you have dry, itchy skin?” (never – always or almost always)

**Internal name:** iqb.poor_circulation

**Publication name:** Poor circulation

**Definition:** “Do you have poor circulation?” (yes, no, not sure)

**Internal name:** ptsd

**Publication name:** Post-traumatic stress disorder

**Definition:** A combination of four questions about PTSD including “Have you ever been diagnosed with post-traumatic stress disorder?” (yes, no, I’m not sure).

**Internal name:** face_age_v2_cc

**Publication name:** Face looks 10 years younger

**Definition:** Female only. Cases: self-report that they appear more than 10 years younger according to others. Controls: do not.

**Internal name:** steroid_meds

**Publication name:** Used steroid medications

**Definition:** A combination of three questions

- “Have you ever taken any of these types of medications?: Prednisone, cortisone, or other steroids” (yes, no, I’m not sure)
- “Have you ever taken these medications?: Prednisone or other steroids” (yes, no, I’m not sure)
- “Have you ever taken an oral corticosteroid medication, such as prednisone or prednisolone?” (yes, no, I’m not sure)

**Internal name:** iqb.agoraphobia

**Publication name:** Agoraphobia

**Definition:** “Do you have agoraphobia (fear of public places, crowds, or open spaces)?” (yes, no, I’m not sure)

**Internal name:** cataract

**Publication name:** Cataract

**Definition:** Cases have been diagnosed with or treated for cataracts. Controls have not been diagnosed with or treated for cataracts.

**Internal name:** farsightedness

**Publication name:** Farsighted

**Definition:** A combination of two questions about being farsighted, including “Are you farsighted (far objects are clear, near objects are blurry)?” (yes, no, I’m not sure)

**Internal name:** liposuction

**Publication name:** Liposuction

**Definition:** Cases: answered “yes” to at least one of several questions pertaining to having had liposuction

**Internal name:** iqb.dry_eyes_treatment

**Publication name:** Dry eyes treatment

**Definition:** “On average, how often do you use some form of treatment for dry eyes, including over-the-counter remedies?” (never – a few times a day)

**Internal name:** years_smoked

**Publication name:** Years smoked (smokers only)

**Definition:** Total number of years smoked, either now or in the past. <1 year scored as 1/2

**Internal name:** does_things_efficiently

**Publication name:** Does things efficiently

**Definition:** I am someone who does things efficiently (strongly disagree – strongly agree)

**Internal name:** lumbar_spine_surgery

**Publication name:** Lumbar spine surgery

**Definition:** “Have you ever had lumbar spine surgery?” (yes, no, I don't know)

**Internal name:** cosmetic_surgery

**Publication name:** Cosmetic surgery

**Definition:** History of cosmetic surgery, including botox for wrinkles, facelift, facial implants, filler injections for wrinkles, laser treatment for skin aging

**Internal name:** iqb.acrophobia

**Publication name:** Fear of heights

**Definition:** “Do you have acrophobia (fear of heights)?” (yes, no, I’m not sure)

**Internal name:** iqb.toenail_fungus

**Publication name:** Toenail fungus

**Definition:** “Have you ever been diagnosed with toenail fungus?” (yes, no, I’m not sure)

**Internal name:** iqb.fresh_cucumbers_yummy

**Publication name:** Enjoys fresh cucumbers

**Definition:** IQB question: “Do you like the taste of fresh cucumbers?” (yes, no, I’m not sure)

**Internal name:** iqb.learn_language_as_adult

**Publication name:** Learned a language as an adult

**Definition:** “Have you learned a foreign language as an adult (age 18 and above)?” (yes, no, not sure)

**Internal name:** iqb.breakfast_frequency

**Publication name:** Breakfast frequency

**Definition:** “During a typical week, how often do you eat breakfast?” (never or almost never – 6 or more times a week)

**Internal name:** gerd_broad

**Publication name:** Gastro-esophageal reflux disease

**Definition:** Cases: answered “yes” to at least one of several questions pertaining to having gastroesophageal reflux disease.

**Internal name:** iqb.desk_clean

**Publication name:** Desk messiness

**Definition:** “Is your desk typically...” (very clean – very messy)

**Internal name:** iqb.apology_prone

**Publication name:** Apology prone

**Definition:** “How often do you apologize for things that are not your fault?” (never – always or almost always)

**Internal name:** seasonal_allergies_strict_controls

**Publication name:** Seasonal allergies

**Definition:** Cases report seasonal allergies on medical history survey, controls report no allergies from allergies, asthma, or medical history survey

**Internal name:** schizophrenia

**Publication name:** Schizophrenia

**Definition:** Combines multiple questions about schizophrenia, including “Have you ever been diagnosed with, or treated for, schizophrenia?” (yes, no, I’m not sure)

**Internal name:** melanoma_dx_or_fh

**Publication name:** Melanoma diagnosis or family history

**Definition:** Aggregates questions about being diagnosed with melanoma or having parents or siblings who have been diagnosed with melanoma. For the former, one of the included questions is “Have you ever been diagnosed with, or treated for, melanoma?” (yes, no, I’m not sure).

**Internal name:** ring_finger_degree_longer

**Publication name:** Ring:index finger length ratio

**Definition:** Relative length of index and ring finger. 0 = index longer, 1 = equal length, 2 = ring longer

**Internal name:** iqb.industry_distance

**Publication name:** Industry distance

**Definition:** “Which of the following best describes how close you live to the nearest industrial facility, factory, or power plant? Your best guess is fine.” (A quarter of a mile or less – 5 miles or more)

**Internal name:** iqb.pee_middle_night

**Publication name:** Frequency of getting up to pee at night

**Definition:** “How often do you typically get up in the middle of the night to pee?” (never – 1 to 3 times a night)

**Internal name:** prostate_enlargement

**Publication name:** Prostate enlargement

**Definition:** A combination of multiple questions, including “Have you ever been diagnosed with, or treated for, enlarged prostate (BPH)?” (yes, no, I’m not sure)

**Internal name:** iqb.forward_splits

**Publication name:** Ever able to do the forward splits

**Definition:** “Have you ever been able to do the forward splits (one leg to the front, and one leg behind)?” (yes, no, I’m not sure)

**Internal name:** iqb.ever_had_allergy_test

**Publication name:** Ever had an allergy test

**Definition:** “Have you ever had an allergy test performed by a medical professional?” (yes, no, I’m not sure)

**Internal name:** iqb.perfect_pitch

**Publication name:** Perfect pitch

**Definition:** “Do you have perfect pitch?” (yes, no, not sure)

**Internal name:** iqb.vitamin_use

**Publication name:** Vitamin use

**Definition:** “Do you take a multivitamin most days?” (yes, no, not sure)

**Internal name:** nonagenarian

**Publication name:** Nonagenarian (self or parent)

**Definition:** Cases: have a nonagenarian parent or is a nonagenarian, controls: have/are neither.

**Internal name:** low_sperm_count

**Publication name:** Low sperm count

**Definition:** From iqb.oligozoospermia and male fertility surveys. Controls say no to iqb and not yes to low sperm count in the fertility survey. The “iqb” is: “Have you ever been told by a doctor that you had a low sperm count (oligozoospermia)?”

**Internal name:** klein_sexual_community_preference_qt

**Publication name:** Klein sexual community score

**Definition:** “In which community do you like to spend your time? In which do you feel most comfortable?” A seven-point scale from “heterosexual only” to “homosexual only”.

**Internal name:** iqb.resting_heart_rate

**Publication name:** Resting heart rate

**Definition:** “What is your resting heart rate (heart rate while sitting still)?” (Fewer than 50 beats per minute – 90 or more beats per minute)

**Internal name:** LASIK

**Publication name:** LASIK

**Definition:** Combines multiple questions about LASIK, including “Have you ever undergone LASIK eye surgery?” (yes, no, I’m not sure)

**Internal name:** number_family_members_cancer

**Publication name:** Number of family members with cancer

**Definition:** Number of reported family members (up to second degree) with cancer

**Internal name:** iqb.supertaster

**Publication name:** Supertaster

**Definition:** IQB question: “Are you a supertaster (someone who is unusually sensitive to certain flavors in food), as confirmed by taste strips?” (yes, no, I’m not sure)

**Internal name:** iqb.ear_plugs_to_sleep

**Publication name:** Uses earplugs to sleep

**Definition:** “How often do you wear ear plugs to help you get to sleep?” (never or almost never – always or almost always)

**Internal name:** motionsick_combined

**Publication name:** Motionsickness frequency

**Definition:** Motionsick, from 0=never to 9=frequent on cars/boats/planes. Sum of scores from 0-3 for cars, boats, and planes.

**Internal name:** male_hair_loss

**Publication name:** Male hair loss

**Definition:** A combination of multiple questions, restricted to males only, pertaining to hair loss including “Have you experienced hair loss or thinning of the hair on your head?” (yes, no, I’m not sure)

**Internal name:** dress_current

**Publication name:** What colours are “the dress”

**Definition:** This question refers to a viral internet phenomenon know as “the dress”. Research participants were shown a photo of “the dress” and asked: “What colors do you currently see in this dress?”. Cases answered “white and gold”, controls answered “blue and black”.

**Internal name:** iqb.cry_cutting_onions

**Publication name:** Cry whilst cutting onions

**Definition:** “How much do your eyes water when cutting onions?” (not at all – a great deal)

**Internal name:** iqb.head_injury_concussion

**Publication name:** Concussion

**Definition:** “Have you ever had a head injury or concussion? Please include injuries that may have occurred during sporting activities, from falls, violence, car accidents, or other accidents, both during your childhood and adulthood.” (yes, no, I’m not sure).

**Internal name:** carpal_tunnel

**Publication name:** Carpal tunnel syndrome

**Definition:** A combination of two survey questions, including “Have you ever been diagnosed with carpal tunnel syndrome?” (yes, no, I’m not sure)

**Internal name:** raynauds

**Publication name:** Raynaud’s

**Definition:** A combination of two questions including “Have you ever been diagnosed with Raynaud's disease or syndrome?” (yes, no, I’m not sure)

**Internal name:** angina

**Publication name:** Angina

**Definition:** Cases: answered “yes” to at least one of several questions pertaining to having angina

**Internal name:** joint_replacement

**Publication name:** Joint replacement

**Definition:** “Have you ever had any of the following bone or joint surgeries?: joint replacement” (yes, no, I don’t know)

**Internal name:** excess_hair

**Publication name:** Excess hair (female only)

**Definition:** “Do you have excess hair growth on your upper lip, chin, lower abdomen, or inner thighs? (restricted to females only)” (yes, no, I’m not sure)

**Internal name:** iqb.whistle

**Publication name:** Can whistle

**Definition:** “Can you whistle?” (yes, no, not sure)

**Internal name:** squamous_cell_carcinoma

**Publication name:** Squamous cell carcinoma

**Definition:** An aggregation of many questions about SCC, including “Have you ever been diagnosed with, or treated for, squamous cell carcinoma?” (yes, no, I’m not sure)

**Internal name:** is_generally_trusting

**Publication name:** Is generally trusting

**Definition:** “I am someone who is generally trusting” (strongly disagree – strongly agree)

**Internal name:** kidney_stones

**Publication name:** Kidney stones

**Definition:** Combines multiple questions, including “Have you ever been diagnosed with, or treated for, kidney stones?” (yes, no, I’m not sure)

**Internal name:** iqb.cartwheel

**Publication name:** Cartwheel

**Definition:** “Have you ever been able to do a cartwheel?” (yes, no, I’m not sure)

**Internal name:** iqb.sweat_while_sleeping

**Publication name:** Sweats while asleep

**Definition:** “Even when sleeping at room temperature, do you sweat a noticeable amount while asleep?” (yes, no, I’m not sure)

**Internal name:** azoospermia

**Publication name:** Azoospermia

**Definition:** “Have you ever been told by a doctor that you had no sperm in your ejaculate (azoospermia)?” (yes, no, I’m not sure)

**Internal name:** iqb.fish_frequency

**Publication name:** Frequency of eating fish

**Definition:** “In a typical week, how often do you eat fish or shellfish?” (not at all – several times a day)

**Internal name:** iqb.feet_different_sizes

**Publication name:** Feet are different sizes

**Definition:** “Is one of your feet a different shoe size from the other?” (yes, no, I’m not sure)

**Internal name:** iqb.malaria_meds

**Publication name:** Malaria medication

**Definition:** “Have you ever taken medication to prevent malaria?” (yes, no, I’m not sure)

**Internal name:** likes_to_cooperate_with_others

**Publication name:** Likes to cooperate with others

**Definition:** “I am someone who likes to cooperate with others” (strongly disagree – strongly agree)

**Internal name:** iqb.wiggle_ears

**Publication name:** Can wiggle ears

**Definition:** “Can you wiggle your ears?” (yes, no, I’m not sure)

**Internal name:** iqb.birth_order

**Publication name:** Birth order

**Definition:** “Which best describes you?” (only child, oldest child, middle child, youngest child, other)

**Internal name:** iqb.sweaty_palms

**Publication name:** Sweaty palms

**Definition:** “Do you typically have sweaty palms?” (yes, no, I’m not sure)

**Internal name:** iqb.fear_of_speaking

**Publication name:** Fear of public speaking

**Definition:** “Do you have a fear of public speaking?” (yes, no, I’m not sure)

**Internal name:** chin_dimple

**Publication name:** Chin dimple

**Definition:** “Do you have a line or indentation (dimple) in the middle of your chin?”

**Internal name:** HIP_urinary_condition_type_SUI

**Publication name:** Stress urinary incontinence

**Definition:** “What type of urinary condition were you diagnosed with most recently?” Cases selected “Stress urinary incontinence (SUI)”, controls did not. From the Mayo Clinic: “Urinary incontinence is the unintentional loss of urine. Stress incontinence happens when physical movement or activity — such as coughing, sneezing, running or heavy lifting — puts pressure (stress) on your bladder. Stress incontinence is not related to psychological stress.”

**Internal name:** HIP_IBS_type_RomeIII_IBS_C

**Publication name:** Constipation-predominant irritable bowel syndrome

**Definition:** Individuals who indicated that they had been diagnosed with IBS were then asked: “What type of irritable bowel syndrome (IBS) were you diagnosed with?” (IBS with predominantly loose or watery stool, IBS with predominantly hard or lumpy stool, IBS with mixed diarrhea and constipation, none of these types, not sure). Cases are individuals who responded with “hard or lumpy”, controls were people with or without IBS.

**Internal name:** iqb.daily_aspirin

**Publication name:** Takes daily aspirin

**Definition:** “Do you take an aspirin every day?” (yes, no, I’m not sure)

**Internal name:** iqb.dry_eyes_procedure

**Publication name:** Dry eyes procedure

**Definition:** “Have you ever undergone a procedure to treat dry eyes?” (yes, no, I’m not sure)

**Internal name:** iqb.pesticide_use

**Publication name:** Current pesticides on home/garden/pet

**Definition:** “In a typical month are any pesticides, including herbicides (to kill weeds), fungicides (to kill fungus/mold), insecticides (to kill insects), rodenticides (to kill rats/mice), or fumigants (gas used to kill insects or fungus or plants) used in your home and garden, or on your pet?” (yes, no, I’m not sure)

**Internal name:** iqb.social_support

**Publication name:** Social support

**Definition:** “If something goes wrong in your life, do you feel that you have someone to turn to?” (yes, no, I’m not sure)

**Internal name:** brain_cancer_dx_or_fh

**Publication name:** Brain cancer diagnosis or family history

**Definition:** This combines parent and sibling diagnoses from three sources: the old cancer_men / cancer_women, cancer_family_hx, and cancer_2016. These all have somewhat different structure so it is tricky to match things up. Cases have a diagnosis of brain cancer or a family history of brain cancer. Controls have neither.

**Internal name:** hernia_repair

**Publication name:** Hernia repair surgery

**Definition:** “Have you ever had any of the following gastrointestinal surgeries?: hernia repair” (yes, no, I don’t know)

**Internal name:** iqb.mild_cognitive_imp_fh

**Publication name:** Family with MCI

**Definition:** “Have any of your grandparents, parents, brothers, sisters, aunts, or uncles ever been diagnosed with mild cognitive impairment (MCI)?” (yes, no, I’m not sure)

**Internal name:** iqb.spicy_food_liking

**Publication name:** Enjoys spicy food

**Definition:** “How much do you like spicy hot food?” (not at all – a great deal)
